# Inconsistencies among European Union Pharmaceutical Regulator Safety Communications: A Cross-Country Comparison

**DOI:** 10.1371/journal.pone.0109100

**Published:** 2014-10-21

**Authors:** Jean-David Zeitoun, Jérémie H. Lefèvre, Nicholas Downing, Henri Bergeron, Joseph S. Ross

**Affiliations:** 1 Sciences Po, Paris, France; 2 Department of Gastroenterology and Nutrition, Saint-Antoine Hospital, APHP, Paris, France; 3 Department of Proctology, Deaconesses Hospital, Paris, France; 4 Department of Digestive and General Surgery, Saint-Antoine Hospital, APHP, Paris, France; 5 University Paris VI, Paris, France; 6 Yale University School of Medicine, New Haven, Connecticut, United States of America; 7 Senior Research Fellow at the National Centre for Scientific Research (CNRS), Paris, France; 8 Section of General Internal Medicine and the Robert Wood Johnson Clinical Scholars Program, Department of Internal Medicine, Yale University School of Medicine, and the Center for Outcomes Research and Evaluation, Yale–New Haven Hospital, New Haven, Connecticut, United States of America; University of British Columbia, Canada

## Abstract

**Background:**

The European Medicines Agency (EMA) and national regulators share the responsibility to communicate to healthcare providers postmarketing safety events but little is known about the consistency of this process. We aimed to compare public availability of safety-related communications and drug withdrawals from the EMA and European Union member countries for novel medicines.

**Methods and Findings:**

We performed a cross-sectional analysis using public Dear Healthcare Professional Communications (DHPCs) for all novel medicines authorized between 2001 and 2010 by the EMA and available for use in France, Netherlands, Spain, and the United Kingdom. Between 2001 and 2010, the EMA approved 185 novel medicines. DHPCs could not be ascertained for the EMA. Among the 4 national regulators, as of April 30, 2013, at least one safety DHPC or withdrawal occurred for 53 (28.6%) medicines, totaling 90 DHPCs and 5 withdrawals. Among these 53 medicines, all 4 national agencies issued at least one communication for 17 (32.1%), three of the four for 25 (47.2%), two of the four for 6 (11.3%), and one of the four for 5 (9.4%). Five drugs were reported to be withdrawn, three by all four countries, one by three and one by two. Among the 95 DHPCs and withdrawals, 20 (21.1%) were issued by all 4 national regulators, 37 (38.9%) by 3 of the 4, 22 (23.2%) by 2 of the 4, and 16 (16.8%) by one. Consistency of making publicly available all identified safety DHPC or withdrawal across regulator pairs varied from 33% to 73% agreement.

**Conclusions:**

Safety communications were not made publicly available by the EMA. Among the 4 European member countries with national regulators that make DHPCs publicly available since at least 2001, there were substantial inconsistencies in safety communications for novel medicines. The impact of those inconsistencies in terms of public health remains to be determined.

## Introduction

The use of nearly all medical therapies carries both the potential for patient benefit and risk, and this is especially true for pharmaceutical products. The so called “life-cycle approach” to drug evaluation, wherein benefits and risks are assessed not only during the pre-market drug development period, but also throughout the post-market “life” of the drug, is currently being championed by leading regulators such as the U.S. Food and Drug Administration (FDA) [1], [2] and the European Medicines Agency (EMA) [3]. But this emerging paradigm is contingent on an effective post-marketing surveillance and communication system for safety signals, so that physicians and patients are updated with relevant contemporary information. Safety risks have been communicated to healthcare providers and the public for decades [4], however, despite their promise, they currently do not have a principal role in a life cycle approach of drug evaluation. The effectiveness and integrity of this communication system is chiefly the responsibility of regulatory agencies.

In Europe, the EMA is responsible for approving the vast majority of drugs but post-marketing safety surveillance is performed by both the EMA and national regulatory agencies (http://www.ema.europa.eu/ema/index.jsp?curl=pages/regulation/general/general_content_000258.jsp&mid=WC0b01ac05800241de). When a safety signal emerges, agencies are able to communicate publicly about potentially unsafe drugs in several ways. Most commonly, a Direct Healthcare Professional Communication (DHPC), so-called “Dear doctor letter”, is issued, the content of which should be agreed by the manufacturer of the drug and by the agency prior to their dissemination. The DHPC is then sent out to healthcare providers in those countries where there is a need or a concern as per agreement with the competent authority, i.e. most frequently the national regulatory agency. Regulators also retain the authority to revoke marketing authorization due to post-marketing safety concerns, effectively withdrawing the drug from the market. As the EMA and national agencies seem to share some regulatory responsibilities, very little is known about how national agencies disseminate DHPCs issued through the EMA or whether these national agencies issue drug safety communications independently and the consequent consistency of these communications across the European Union (EU).

If drug safety communications are not consistently made publicly available by local regulators after the EMA first acts to call public attention to a safety concern, patients and healthcare professionals are not being provided with complete and necessary information to guide their decisions to use or prescribe a medication. Similarly, if local regulators inconsistently make safety communications public, conveying some but not others, it may create public confusion given the close proximity and communication between EU member countries. Accordingly, our research objective was to assess the consistency of DHPCs publicly communicated among countries under the jurisdiction of the EMA, with respect to both the availability of DHPCs and their timing.

## Methods

### Novel Therapeutic Sample

For purposes of studying consistency in safety communications across multiple European regulatory agencies, we studied a sample of novel therapeutic agents approved by the EMA between January 1, 2001 and December 31, 2010, by the Centralized Authorization Procedure of the EMA, that had been developed for prior work [5]. In brief, we identified all novel therapeutic agents approved during this period, including small molecules and biologics. We excluded reformulations, combinations therapies, and nontherapeutic agents, such as radiographic dye.

### European Regulatory Agencies Sample

To determine which European regulatory agencies should be included for comparison, we first identified the ten countries of the EU with the highest total drug sales in 2011 using IMS Health data (**Table 1**; information provided at personal request by Loïc Lebrun from IMS Health on April 2, 2013). We made such a choice because we anticipated that those countries would be more relevant for a regulatory study and in particular they would have resources to implement an effective communicating infrastructure to healthcare providers. Next, for each country, the national regulatory agency for human medicines was identified through the EMA's website, via the “partners and networks” section, and we determined whether each agency maintained its own website that included a dedicated section for safety information, publicly posting DHPCs as of 2001, the beginning of our novel therapeutic sample period. We limited our study to agencies that maintain a website containing a history of issued DHPCs, as this was the only mean of systematically examining past communications. Of the 10 identified agencies, four had been providing DHPCs on-line since 2001: France, Netherlands, Spain, and United Kingdom. Germany and Sweden only began providing DHPCs on-line after 2009, Belgium in 2011; Italy, Greece, and Poland do not currently make DHPCs available via the internet.

**Table 1:** Top ten European Union member countries, ranked by pharmaceutical expenditures, and public availability of Direct Healthcare Professional Communications (including drug withdrawals).

European Pharmaceutical Market	Public Availability of Direct Healthcare Professional Communications
Germany	From September, 2009
France	From December, 1998
Italy	Unavailable
United-Kingdom	From April, 1999
Spain	From November, 1999
Poland	Unavailable
Belgium	From January, 2011
Netherlands	From November, 1998
Greece	Unavailable
Sweden	From January, 2009

Source: IMS Health.

### EMA DHPC Search Strategy

Before searching each national agency website, we systematically searched the EMA website in order to determine whether a DHPC had been issued for each of the novel medicines included in our sample from January 2001 to April 2013, using both the brand name and the name of the molecule. We initially searched within the “Find Medicines” and “Human Medicines” sections of the website, wherein the EMA maintains European Public Assessment Reports, summarizing premarketing scientific discussion and post-approval information. However, no systematic information on DHPCs issued by the EMA was found, or elsewhere within other sections of the website. The lack of full availability of these communications was confirmed directly with EMA representatives (personal communication with the Information Department, March, 2013). Therefore, DHPCs were not made publicly available by the EMA and could not be studied.

### European Regulatory Agencies DHPC Search Strategy

For each national agency, we systematically performed a search of their website in order to determine whether a DHPC, or Dear Doctor Letter, had been made publicly available for each of the novel medicines included in our sample between January 2001 to April 2013, using both the brand(s) name(s) and the name of the molecule. Each agency required a slightly modified search strategy, tailored to the specifics of its website.

For the French National Agency (*Agence nationale de sécurité du médicament et des produits de santé*, National Agency for the Safety of Medicine and Healthcare Products, www.ansm.sante.fr), we searched on the “information” section, then on the “safety information” section, and then on the “letters to healthcare professionals” providing a list of DHPCs and withdrawals issued since December, 1998.

For the Dutch Agency (Medicines Evaluation Board, www.cbg-meb.nl), we searched on the “human medicines” section, then on the “pharmacovigilance” section, and then on the “Dear Healthcare Professional Communications” section providing the list of all DHPCs and withdrawals issued since November, 1998.

For the Spanish Agency (Spanish Agency for Medicines and Health Products, www.aemps.gob.es), we searched on the “medicines for human use” section, then on the “safety warnings” section providing a list of DHPCs and withdrawals issued since November, 1999.

Finally, for the United-Kingdom Agency (Medicines and Healthcare Products Regulatory Agency, www.mhra.gov.uk), we searched on the “safety information” section, then on the “safety warnings, alerts and recalls” section, and then on the “safety warnings and messages for medicines” section providing a list of DHPCs and withdrawals issued since April, 1999. From July, 2006, DHPCs issued by the MHRA are systematically gathered in a subsection at the end of each month.

### Identification of DHPCs and Withdrawals

For every DHPC or withdrawal identified during our search, we determined whether the DHPC was communicating a safety concern or whether the withdrawal was related to a safety issue. For each, the following information was manually abstracted by JDZ: full text of the DHPC or withdrawal (if given) and date(s) of digital issuance (and not the date stated on the DHPC itself). If necessary, translation of the DHPC was performed using Google Translate (Google, Inc.; Mountain View, CA, USA). DHPCs related to technical problems (dosage, route of administration, quality defects), supply issues, or efficacy issues were not categorized as safety communications and were subsequently excluded. DHPCs communicating both safety and efficacy issues were included.

Each time an inconsistency was identified regarding a DHPC or withdrawal, a manual search was performed through the search engine of the website and using Google to screen for a DHPC inadvertently placed at other locations of the website and to determine whether the product involved was effectively marketed in the country that had not issued a DHPC or withdrawal.

### Statistical Analysis

We used descriptive analysis to characterize each regulatory agency sample, including the proportion of therapeutics for which a safety DHPC or withdrawal was issued and the median number of DHPCs issued per therapeutic.

We used percent agreement and kappa statistics to compare whether national agencies issued a safety DHPC or withdrawal for all novel therapeutics included in our sample, as well as for products in which one or more safety events had been issued. If one national regulator had issued a DHPC or withdrawal but the product was not marketed in another national market that had not, the regulators were considered to be ‘in agreement’.

The median test (Mood's test) was used for analysis of delay between the first and the last issue for any event (DHPC and withdrawal) reported by two or more national regulatory agencies. For therapeutics for which DHPCs/withdrawals were issued in multiple countries, we calculated the time difference between communications from the two (or more) regulatory agencies.

All tests were two sided, with the significance level set at 0.05. Analyses were performed using JMP, Version 9 (SAS Institute Inc.; Cary, NC, USA).

## Results

From January 1, 2001 to December 31, 2010, 185 novel therapeutic agents meeting the inclusion criteria were approved by the EMA, among which 53 (28.6%) received at least one DHPC safety communication or were withdrawn in at least one of the 4 countries from January 1, 2001 to April 30, 2013. Among these, there was a total of 95 different safety communications, including 5 withdrawals. Overall, one safety communication was issued for 30 (56.6%) medicines, two for 11 (20.8%) medicines, three for 7 (13.2%), and four or more for 5 (9.4%).

**Figure 1:**
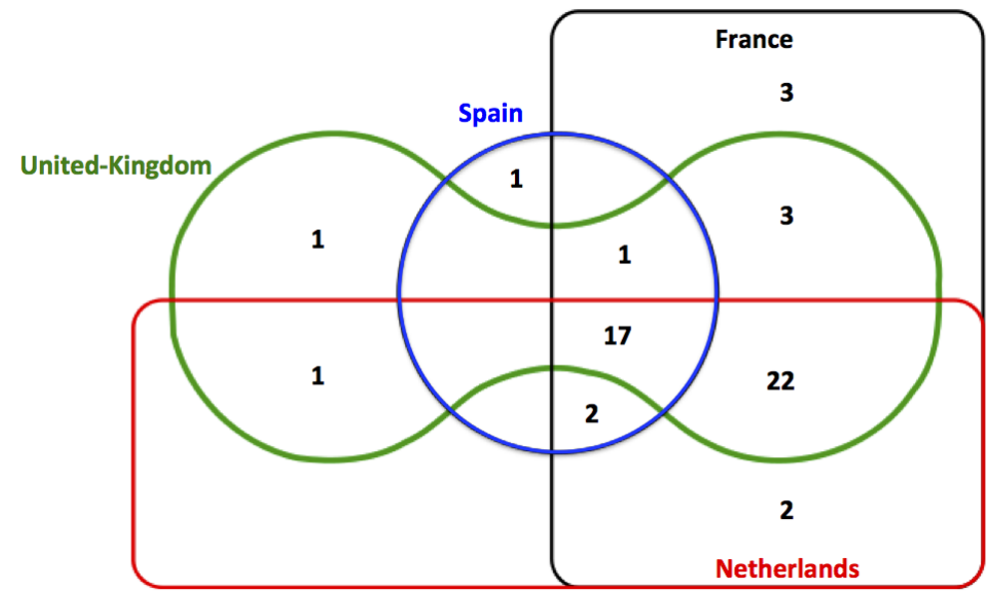
Venn diagram demonstrating whether any safety communication was reported by the four national regulatory agencies for 53 novel medicines approved between 2001 and 2010.

Among the 53 novel medicines for which at least one safety communication or withdrawal was issued, all 4 national regulatory agencies issued at least one communication for 17 (32.1%), three of the four for 25 (47.2%), two of the four for 6 (11.3%), and only one of the four for 5 (9.4%) (Figure 1). Overall, the French regulator issued at least one safety communication for 50 (28.3%) of the medicines in our sample, the Dutch regulator 44 (23.9%), the Spanish regulator 21 (11.4%), and the United-Kingdom regulator 45 (24.5%).

Five drugs were withdrawn after approval, three by all four countries (Thelin/Sitaxenten sodium; Raptiva/efalizumab; Acomplia/rimonabant), one withdrawal was reported by three countries but was not marketed in the fourth (Tredaptive/laropiprant suspended by Spain, UK and Netherlands, not marketed in France), and one was reported by only two countries (Xigris/drotrecogin alfa suspended by France and UK). For Sitaxenten sodium, one DHPC was issued by one country (Spain) preceding withdrawal approximately one month later. For efalizumab, two DHPCs were issued preceding withdrawal, the first by France related to peripheral neuropathy and a second related to progressive multifocal leukoencephalopathy by three countries; the medicine was withdrawn approximately three months later. For rimonabant, two DHPCs were issued preceding withdrawal, the first by three countries in July 2007 related to psychiatric effects, the second approximately one year later by all four countries for similar AEs; the medicine was withdrawn approximately three months later. For laropiprant, one DHPC was issued by two countries preceding its withdrawal approximately two weeks later. Finally, for drotrecogin alfa, one DHPC was issued by two countries (France, Netherlands) preceding its withdrawal by two countries (France and UK). Detailed results are presented in the appendix (see Appendix S1).

Consistency of issuing any safety DHPC or withdrawal for medicines across national regulator pairs varied (Table 2, Figure 1). The highest agreement was observed between the national regulators of France and Netherlands (% agreement = 95.7%; kappa = 0.89), whereas the lowest was observed between France and Spain (% agreement = 83.1%; kappa = 0.47). The kappa coefficients and corresponding 95% confidence intervals for country-to-country comparison are detailed in Table 2.

**Table 2:** Consistency of making any safety Direct Healthcare Professional Communications (DHPCs) or withdrawal publicly available for 185 medicines or all 95 identified safety DHPCs or withdrawals across national regulator pairs.

National Regulator Agency Pair		Any Safety DHPC or Withdrawal among all 185 medicines			All 95 Safety DHPCs or Withdrawals Identified among 53 medicines		
		Percent Agreement	Kappa 95% CI		Percent agreement	Kappa 95% CI	
France	Netherlands	95.7%	0.89	[0.81 to 0.96]	72.6%	0.27	[0.08 to 0.46]
France	UK	95.1%	0.87	[0.79 to 0.95]	72.6%	0.17	[−0.05 to 0.36]
Netherlands	UK	95.1%	0.87	[0.78 to 0.95]	68.4%	0.24	[0.04 to 0.45]
Netherlands	Spain	85.2%	0.50	[0.34 to 0.65]	60.6%	0.09	[−0.08 to 0.25]
UK	Spain	84.1%	0.47	[0.32 to 0.63]	37.9%	−0.01	[−0.16 to 0.14]
France	Spain	83.1%	0.47	[0.32 to 0.61]	33.0%	−0.12	[−0.24 to 0.01]

**Note:** CI = Confidence Interval. Guide to Kappa Interpretation [9]: <0, Less than chance agreement; 0.01 to 0.20, Slight agreement; 0.21 to 0.40, Fair agreement; 0.41 to 0.60, Moderate agreement; 0.61 to 0.80: Substantial agreement; and 0.81 to 0.99: Almost perfect agreement.

**Figure 2:**
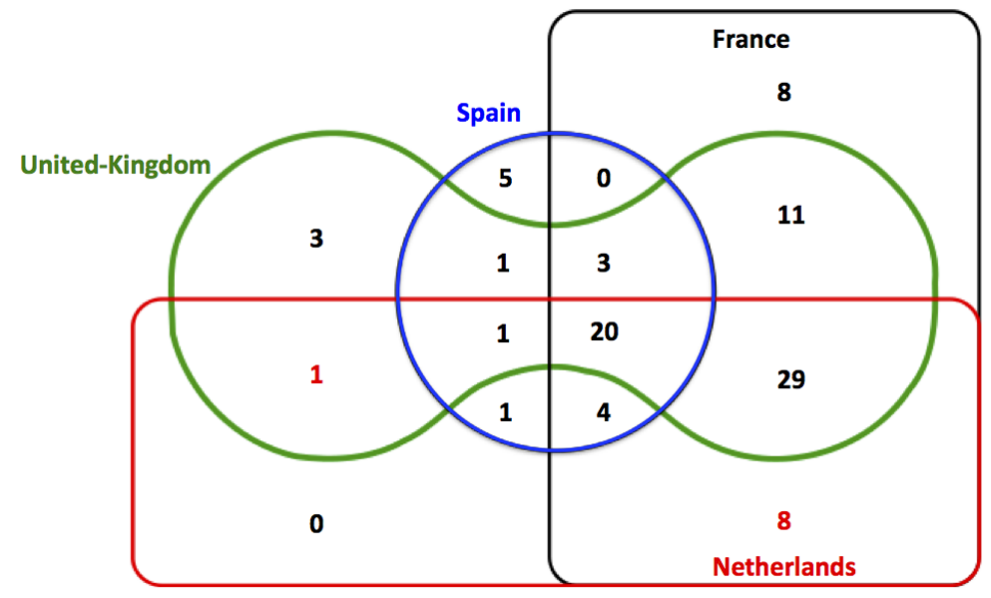
Venn diagram demonstrating whether each of 95 safety communication was reported by the four national regulatory agencies for 53 novel medicines approved between 2001 and 2010.

Among the 95 total safety communications and withdrawals that were issued, 20 (21.1%) were issued by all 4 national regulators, 37 (38.9%) by 3 of the 4, 22 (23.2%) by 2 of the 4, and 16 (16.8%) by only one (Figure 2). Consistency of issuing all identified safety DHPC or withdrawal for medicines across national regulator pairs also varied. The highest agreement was observed between the national regulators of France and Netherlands (% agreement = 72.6%; kappa = 0.27), whereas the lowest was observed between France and Spain (% agreement = 33.0%; kappa = −0.12).

Among the 79 safety DHPCs and withdrawals that were issued by at least two regulators, the median time difference was 13.0 days (Interquartile Range [IQR]: 7–27). The greater the number of regulators that issued DHPCs or withdrawals, the longer the median time between the first and last communication; median difference when two regulators issued safety communications was 9 days (IQR = 3.5-1), three regulators was 15 days (IQR = 7–28), and four regulators was 21 days (IQR = 3–30) (p = 0.03).

**Figure 3:**
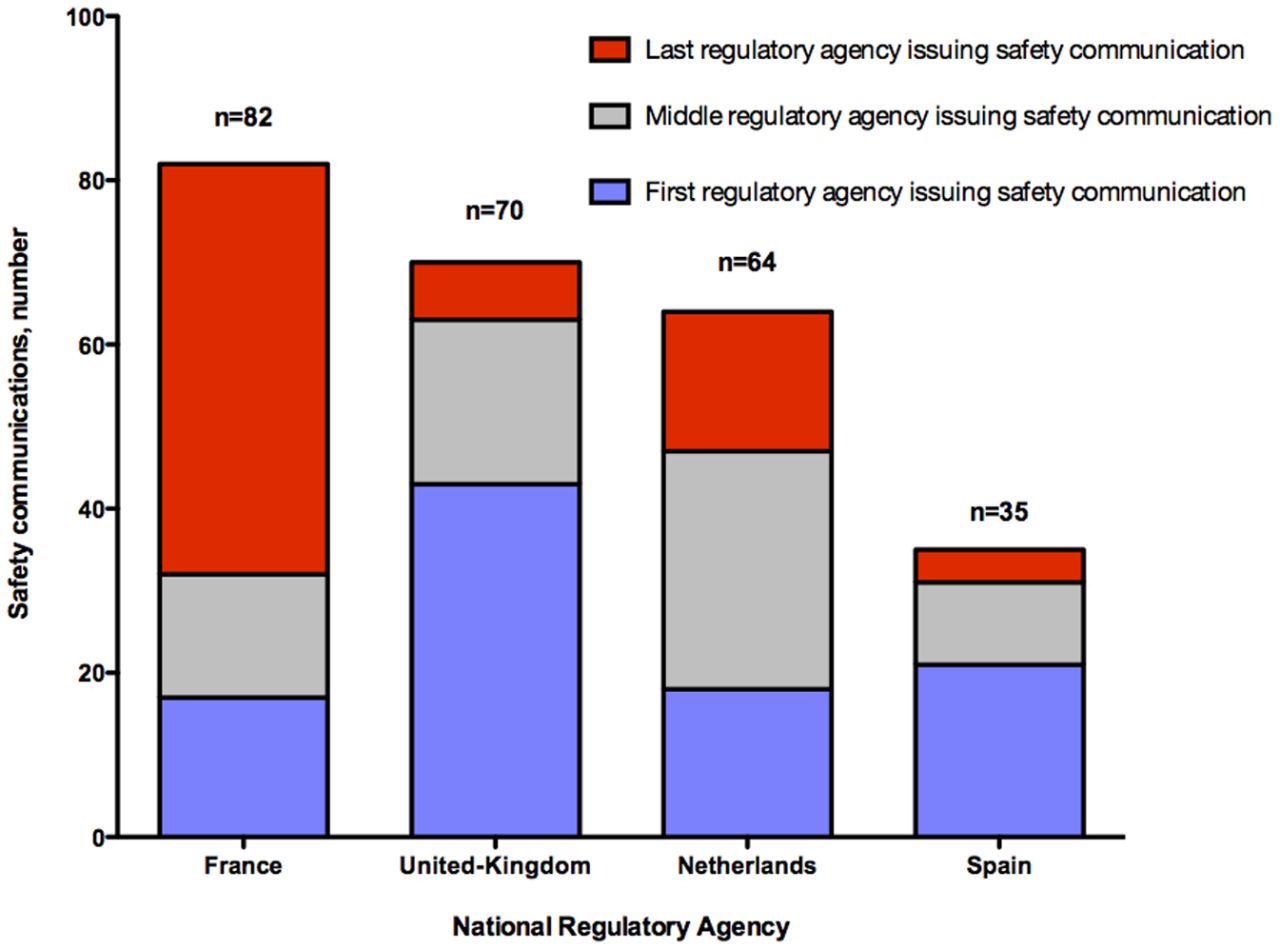
Number and timing of 95 safety communications reported for 53 novel medicines approved between 2001 and 2010 among four national regulatory agencies.

The Netherlands and United Kingdom regulators each issued at least one DHPC or withdrawal for 48 medicines and the median time difference in these communications was 7 days (IQR = 2–13; Table 3). In contrast, French and Spanish regulators each issued at least one DHPC or withdrawal for 21 medicines and the median time difference in these communications was 13.5 days (IQR = 11–23). Spain consistently was the first to issue safety communications when compared to the other individual agencies, whereas France was consistently last, with time differing on average from 9 to 14 days (Table 3, Figure 3).

**Table 3:** Timing of making safety Direct Healthcare Professional Communications (DHPCs) or withdrawals publicly available among medicines approved by the European Medicines Agency between 2001 and 2010 between national regulator pairs.

National Regulator Agency Pair		Common Safety Communications, No.	National Regulatory Agency First Issuing Communication, No. (%)	Difference in Issuance of Safety Communications Between National Regulatory Agencies (Days)	
				Median	InterQuartile Range
France	UK	63	UK: 52 (82.5%)	12	7–25.5
France	Netherlands	60	Netherlands: 38 (63.3%)	9	2.5–16
Netherlands	UK	52	UK: 34 (65.4%)	5	1–12.5
France	Spain	26	Spain: 14 (53.8%)	13.5	8–26
UK	Spain	25	Spain: 14 (56%)	7.5	2.25–17.5
Netherlands	Spain	25	Spain: 14 (56%)	7	2–13.5

## Discussion

In our study of all novel therapeutic medicines approved by the EMA from 2001 to 2010, we found that the EMA and European national regulatory agencies do not comprehensively and consistently publicly communicate post-marketing safety concerns. The EMA does not currently display a publicly available list of safety-related DHPCs issued since 2001 that had been transmitted to national regulatory agencies. Among the top 10 highest prescribing EU countries, only 4, France, Netherlands, Spain and the United-Kingdom, currently make DHPCs publicly available since 2001. Some countries like Germany, Belgium or Sweden have only recently begun to display on their website information regarding DHPCs. These findings suggest that patients and physicians from the EU member countries are likely to face difficulties and confusion when trying to obtain official and reliable information about drug safety.

Among the 4 countries that have been making safety-related DHPCs available to the public, we found many discrepancies in the communications, both by therapeutic product and in the specific communications. Many safety events were communicated in few countries but not all, and even when all 4 countries issued a DHPC for a given adverse event, we frequently observed significant delays of public communications between regulators. Of note is the fact that the French regulator was both the one that issued the greater number of DHPCs and the slowest regulator on average to publish them. Whether those two features are linked and should be interpreted as rigorous and thorough, but potentially slow, deserves further study.

There are several ways by which patients and physicians are able to obtain updated information about drug safety, including from the medical literature and directly from product manufacturers. However, information that is communicated directly by regulators under the form of DHPCs is thought to represent the mainstay of pharmacovigilance communication to physicians and some authors have shown that DHPCs had been issued in increasing numbers over the past decade [6]. The accessibility, clarity and reliability of these DHPCs are paramount. If the information they aim to disseminate is not effectively transmitted to healthcare providers, drugs are more likely to be used or prescribed without appropriate caution and likely without fully informing patients of the true risks of the medicine, which has potential implications for public health [7]. We found a relatively high number of safety-related communications within the period of interest, in line with previous reports [8] and in a way reassuring in that it suggests that the system is functioning effectively to communicate safety concerns across EU countries. However, the quantity of pharmacovigilance statements provides only partial reassurance given the observed inconsistencies among the four examined regulators, particularly with respect to the kappa tests for all 95 DHPCs or withdrawals identified as being publicly available for the study period. Estimates indicate that agreement between countries was only slight to fair, with scores below 0.41, the accepted cut-off for moderate agreement [9].

There are several possible reasons for the discrepancy in public safety communications among these four countries. First, national regulators may be overwhelmed by administrative burden, creating inconsistencies in safety communications. Second, national regulators may be working under different safety policies, such that some countries may believe that not all safety events are required to be reported to healthcare providers under the form of a DHPC. Such a choice could be justified by the fact that some adverse events would not be of sufficient interest or by the concern that issuing too many DHPCs could saturate clinicians to appropriately process the communication.

At the very least, the EMA should begin to make publicly available a list of safety-related DHPCs that had been transmitted to national regulatory agencies. While the EMA is presently engaging in a process of comprehensive transparency regarding clinical trial data for medicines that have been or were considered for approval [10], enhancing pharmacovigilance transparency is equally important. It is the responsibility of the EMA to take over the leadership for the improvement of the provision of advice to the safe use of medicines. In this regard, it is worth noting that the U.S. Food and Drug Administration has in recent years committed to a significant endeavor to provide reliable, comprehensive and transparent safety information [1], [2].

There are several limitations to our study. First, our search of DHPCs was limited to the websites of the regulators being studied. We cannot rule out the possibility of a regulator having prepared a DHPC, or even communicating it with physicians or patients, but not indexing it on the regulator's website for public access. However, because we used a systematic search strategy that thoroughly scanned each regulator's public communications and because the purpose of our study was to examine the consistency in public drug safety communications across European regulators, any lack of online reporting can be interpreted as a failure of the pharmacovigilance communication process. Second, we performed manual searches to determine marketing status of all drugs for which a DHPC had been issued by one national regulator and not another. As none of the four national regulators maintain a database of medications that have been approved for use along with current marketing status, we may not have accurately determined marketing status of all relevant medications. Similarly, we performed manual searches of all national regulatory agency websites for DHPCs and withdrawals because no downloadable list of these safety communications was available. It is possible that some DHPCs were not identified. In fact, several adverse events communications were found through Google searches within regulatory agency websites, but were not formally issued as DHPCs. Similarly, the United-Kingdom uses a “Black Triangle List” to bring attention to currently marketed drugs under safety surveillance. But the list of drugs is long and these concerns are not formally communicated to clinicians as DHPCs, limiting their impact to inform physicians and patients. Nevertheless, for both searches, we used a systematic search strategy with discussion and confirmation among multiple investigators. Finally, we could not assess the actual impact of the observed inconsistencies across national regulators in safety communications from a public health perspective. Additional research is needed to examine this issue.

In conclusion, we found that numerous safety-related DHPCs were issued from 2001 to 2013 for all medicines approved by the EMA between 2001 and 2010. However, safety communications were not made publicly available by the EMA. Among the 4 European member countries with national regulatory agencies that make DHPCs publicly available since 2001, there were substantial inconsistencies in making safety communications public for newly authorized medicines. Although the impact of these differences could not be assessed, it raises questions about safety policies and regulatory efficiency of the countries involved and about the possible confusion it could provide among patients and physicians.

## Supporting Information

Appendix S1 (DOCX)
