# Supplementary material for: Inconsistencies among European Union Pharmaceutical Regulator Safety Communications: A Cross-Country Comparison
Source: PLoS One. 2014 Oct 21;9(10):e109100. doi: 10.1371/journal.pone.0109100 (PMC4204813; doi:10.1371/journal.pone.0109100)
Supplement: Appendix S1 — (DOCX) [file pone.0109100.s001.docx]

List of 53 medicines for which publicly available safety communications, Direct Healthcare Professional Communications (DHPCs) and withdrawals) were identified from any of the regulators after approval, the type of communication, and the safety concern described.

**Notes:** W, drug withdrawal; X, DHPC; and n/a, non-applicable as medicine was never marketed in the country.

|  |  |  | **Regulator Public Communication of Safety** | | | |
| --- | --- | --- | --- | --- | --- | --- |
| **Drug (name/common name)** | **Type of safety communication** | **Type of adverse event** | **France** | **United-Kingdom** | **Netherlands** | **Spain** |
| Abilify/aripiprazole | DHPC | Stroke | X |  |  |  |
| Acomplia/rimonabant | Withdrawal | Psychiatric effects | W | W | W | W |
| Adenuric/febuxostat | DHPC | Hypersensitivity reactions | X | X | X |  |
| Aptivus/tipranavir | DHPC | Intracranial hemorrhage | X | X | X | X |
| Arixtra/fondaparinux sodium | DHPC | Bleeding events | X |  |  |  |
| Avastin/bevacizumab | DHPC | Tracheo-esophageal fistula | X | X | X |  |
| Brinavess/vernakalant hydrochloride | DHPC | Risk of severe hypotension | X | X | X |  |
| Champix/varenicline | DHPC | Depression and cardiovascular events |  |  |  | X |
| Cubicin/daptomycin | DHPC | Eosinophilic pneumonia | X | X | X |  |
| Dynastat/parecoxib | DHPC | Cardiovascular and skin toxicity | X |  | X | X |
| Efient/prasugrel | DHPC | Hypersensitivity | X | X | X |  |
| Enviage/aliskiren | DHPC | New Contraindication | X | X | X | X |
| Exjade/deferasirox | DHPC | Liver and renal toxicity and gastrointestinal hemorrhage |  | X |  |  |
| Glivec/imatinib | DHPC | Preclinical carcinogenicity and heart failure | X | X | X |  |
| InductOs/dibotermin alfa | DHPC | Postoperative edema and infection | X | X | X |  |
| Intelence/etravirine | DHPC | Drug Reaction with Eosinophilia and Systemic Symptoms | X | X | X | X |
| Kepivance/palifermin | DHPC | Restricted indication | X | X | X |  |
| Ketek/telithromycin | DHPC | Restriction use and new contraindication | X | X |  | X |
| Kineret/anakinra | DHPC | Severe infection | X |  | X |  |
| MabCampath/alemtuzumab | DHPC | Infection-related deaths in clinical trials | X | X | X | X |
| Mimpara/cinacalcet | DHPC | Fatal cases of hypocalcaemia in pediatric trials | X | X |  |  |
| Multaq/dronedarone | DHPC | Liver injury | X | X | X | X |
| Nplate/romiplostim | DHPC | Warning of use in hepatic failure and risk of leukemia | X | X | X | X |
| Onglyza/saxagliptin | DHPC | Hypersensitivity and acute pancreatitis | X | X |  |  |
| Tredaptive/laropiprant / nicotinic acid | Withdrawal | Increased mortality in clinical trials | n/a | W | W | W |
| Pradaxa/dabigatran etexilate mesilate | DHPC | New contraindication and need to assess renal function | X | X | X | X |
| Prolia/denosumab | DHPC | Atypical femoral fracture | X | X | X |  |
| Protelos/strontium ranelate | DHPC | Drug Reaction with Eosinophilia and Systemic Symptoms and thrombosis | X | X | X | X |
| Protopic/tacrolimus | DHPC | Increased risk of malignancies | X | X | X | X |
| Raptiva/efalizumab | Withdrawal | Peripheral neuropathy and Progressive Multifocal Leukoencephalopathy | W | W | W | W |
| Relistor/methylnaltrexone bromide | DHPC | Gastrointestinal perforation | X | X | X |  |
| Revlimid/lenalidomide | DHPC | Thrombosis, malignancy and hepatic injury | X | X | X |  |
| RoActemra/tocilizumab | DHPC | Hypersensitivity reaction | X | X | X |  |
| Sebivo/telbivudine | DHPC | Peripheral neuropathy | X | X | X |  |
| Sprycel/dasatinib | DHPC | Pulmonary hypertension | X | X | X |  |
| Sutent/sunitinib | DHPC | Jaw osteonecrosis | X | X |  |  |
| Tarceva/erlotinib | DHPC | Gastrointestinal perforation |  | X | X |  |
| Thalidomide Celgene/thalidomide | DHPC | Arterial thrombosis | X | X | X | X |
| Thelin/sitaxentan sodium | Withdrawal | Hepatotoxicity | W | W | W | W |
| Thymanax/agomelatine | DHPC | Hepatotoxicity | X | X | X |  |
| Torisel/temsirolimus | DHPC | Hypersensitivity and infusion reaction | X | X | X |  |
| Tracleer/bosentan monohydrate | DHPC | Hepatic injury | X |  |  |  |
| Trudexa/adalimumab | DHPC | T-cell hepatosplenic lymphoma | X | X | X |  |
| Tygacil/tigecycline | DHPC | Increased mortality in trials | X | X | X |  |
| Tysabri/natalizumab | DHPC | Hepatic injury and PML | X | X | X | X |
| Vectibix/panitumumab | DHPC | Hypersensitivity reactions and keratitis | X | X | X |  |
| Velcade/bortezomib | DHPC | New contraindications | X |  | X |  |
| Vfend/voriconazole | DHPC | Hepatic injury and risk of carcinoma | X | X | X |  |
| Viread/tenofovir disoproxil fumarate | DHPC | Renal injury | X | X | X | X |
| Volibris/ambrisentan | DHPC | Contraindicated in pulmonary fibrosis | X | X | X | X |
| Xagrid/anagrelide | DHPC | Cardiovascular events | X | X | X |  |
| Xigris/drotrecogin alfa (activated) | Withdrawal | Benefits no longer outweigh risks | W | W | X |  |
| Zavesca/miglustat | DHPC | Colitis and colonic carcinomas in animals | X |  | X |  |
